# Supplementary figures and images for: EXOC1 plays an integral role in spermatogonia pseudopod elongation and spermatocyte stable syncytium formation in mice
Source: eLife. 2021 May 11;10:e59759. doi: 10.7554/eLife.59759 (PMC8112867; doi:10.7554/eLife.59759)

## Slide 1
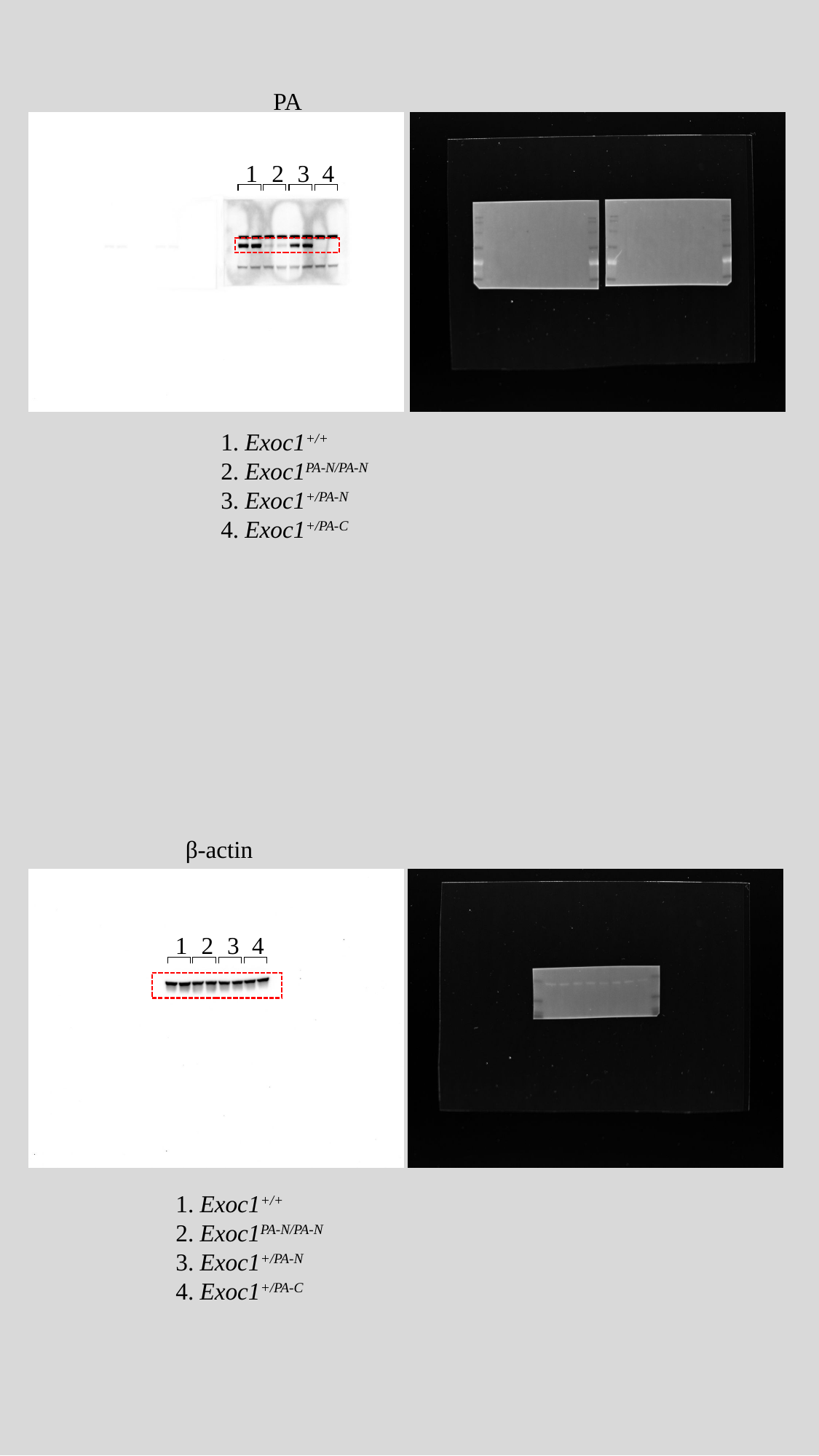

PA
1
2
3
4
1. Exoc1+/+
2. Exoc1PA-N/PA-N
3. Exoc1+/PA-N
4. Exoc1+/PA-C
β-actin
1
2
3
4
1. Exoc1+/+
2. Exoc1PA-N/PA-N
3. Exoc1+/PA-N
4. Exoc1+/PA-C

Supplement: Figure 1—source data 1. [file elife-59759-fig1-data1.pptx]
