# Supplementary material for: EXOC1 plays an integral role in spermatogonia pseudopod elongation and spermatocyte stable syncytium formation in mice
Source: eLife. 2021 May 11;10:e59759. doi: 10.7554/eLife.59759 (PMC8112867; doi:10.7554/eLife.59759)
Supplement: Figure 3—source data 1. [file elife-59759-fig3-data1.pptx]

## Slide 1
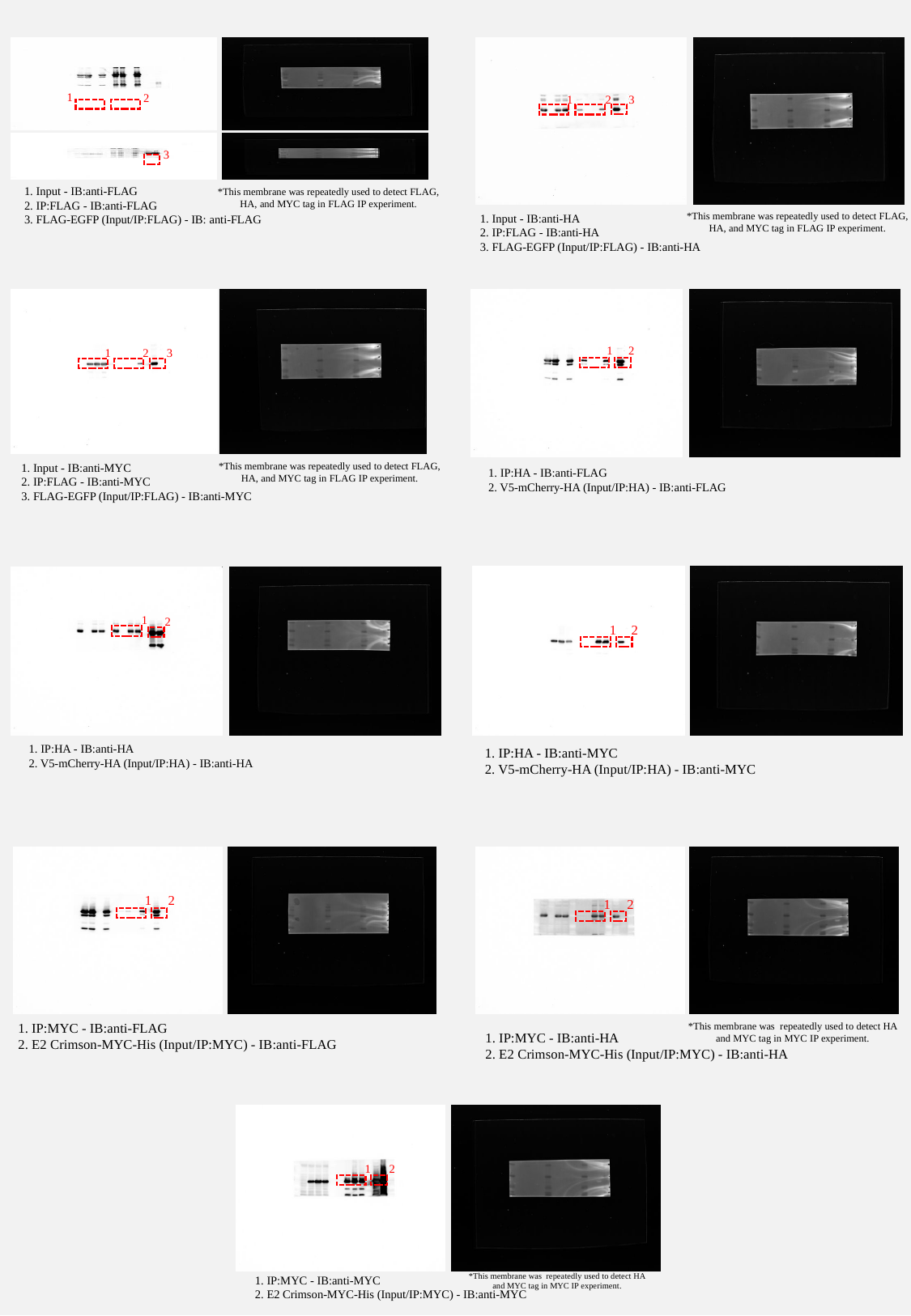

1
2
1
2
3
3
1. Input - IB:anti-FLAG
2. IP:FLAG - IB:anti-FLAG
3. FLAG-EGFP (Input/IP:FLAG) - IB: anti-FLAG
*This membrane was repeatedly used to detect FLAG,
HA, and MYC tag in FLAG IP experiment.
*This membrane was repeatedly used to detect FLAG,
HA, and MYC tag in FLAG IP experiment.
1. Input - IB:anti-HA
2. IP:FLAG - IB:anti-HA
3. FLAG-EGFP (Input/IP:FLAG) - IB:anti-HA
2
1
1
2
3
*This membrane was repeatedly used to detect FLAG,
HA, and MYC tag in FLAG IP experiment.
1. Input - IB:anti-MYC
2. IP:FLAG - IB:anti-MYC
3. FLAG-EGFP (Input/IP:FLAG) - IB:anti-MYC
1. IP:HA - IB:anti-FLAG
2. V5-mCherry-HA (Input/IP:HA) - IB:anti-FLAG
1
2
1
2
1. IP:HA - IB:anti-HA
2. V5-mCherry-HA (Input/IP:HA) - IB:anti-HA
1. IP:HA - IB:anti-MYC
2. V5-mCherry-HA (Input/IP:HA) - IB:anti-MYC
1
2
1
2
*This membrane was repeatedly used to detect HA
and MYC tag in MYC IP experiment.
1. IP:MYC - IB:anti-FLAG
2. E2 Crimson-MYC-His (Input/IP:MYC) - IB:anti-FLAG
1. IP:MYC - IB:anti-HA
2. E2 Crimson-MYC-His (Input/IP:MYC) - IB:anti-HA
1
2
*This membrane was repeatedly used to detect HA
and MYC tag in MYC IP experiment.
1. IP:MYC - IB:anti-MYC
2. E2 Crimson-MYC-His (Input/IP:MYC) - IB:anti-MYC
