# Supplementary material for: EXOC1 plays an integral role in spermatogonia pseudopod elongation and spermatocyte stable syncytium formation in mice
Source: eLife. 2021 May 11;10:e59759. doi: 10.7554/eLife.59759 (PMC8112867; doi:10.7554/eLife.59759)
Supplement: Figure 3—source data 2. [file elife-59759-fig3-data2.pptx]

## Slide 1
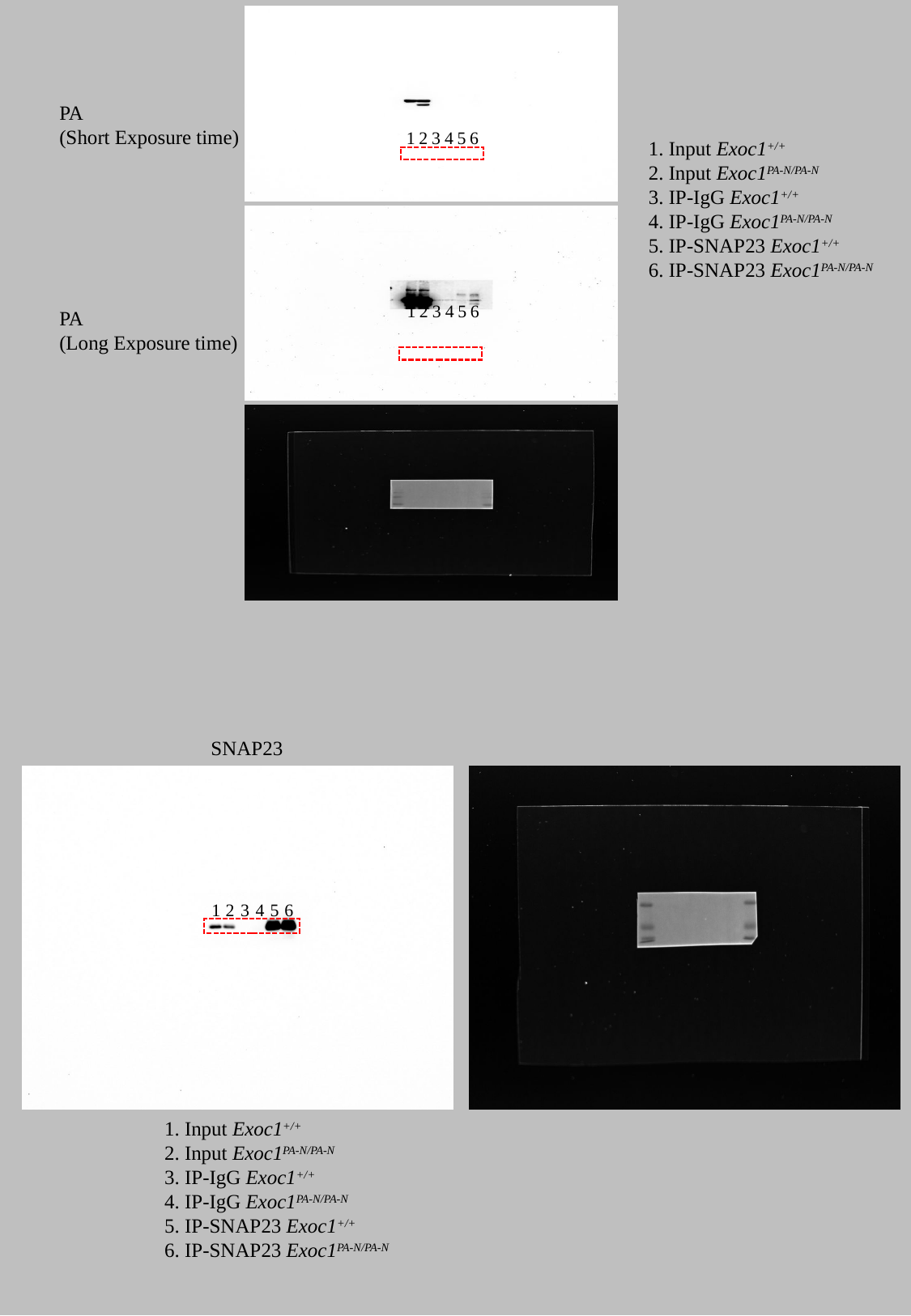

PA
(Short Exposure time)
1
2
3
4
5
6
1. Input Exoc1+/+
2. Input Exoc1PA-N/PA-N
3. IP-IgG Exoc1+/+
4. IP-IgG Exoc1PA-N/PA-N
5. IP-SNAP23 Exoc1+/+
6. IP-SNAP23 Exoc1PA-N/PA-N
1
2
3
4
5
6
PA
(Long Exposure time)
SNAP23
1
2
3
4
5
6
1. Input Exoc1+/+
2. Input Exoc1PA-N/PA-N
3. IP-IgG Exoc1+/+
4. IP-IgG Exoc1PA-N/PA-N
5. IP-SNAP23 Exoc1+/+
6. IP-SNAP23 Exoc1PA-N/PA-N
